# Long non-coding RNA RP11-70C1.3 confers chemoresistance of breast cancer cells through miR-6736-3p/NRP-1 axis

**DOI:** 10.17305/bjbms.2021.5803

**Published:** 2021-06-29

**Authors:** Lansheng Zhang, Xia Zheng, Anqi Shen, Daojin Hua, Panrong Zhu, Caihong Li, Zhengxiang Han

**Affiliations:** 1 Department of Radiotherapy, The Second Affiliated Hospital of Xuzhou Medical University, Xuzhou, China; 2 Department of Oncology, The Affiliated Hospital of Xuzhou Medical University, Xuzhou, China

**Keywords:** Breast cancer, RP11-70C1.3, chemoresistance, NRP-1, miR-6736-3p

## Abstract

Chemoresistance remains a major obstacle for improving the clinical outcome of patients with breast cancer. Recently, long non-coding RNAs (lncRNAs) have been implicated in breast cancer chemoresistance. However, the function and underlying mechanism are still largely unknown. Using lncRNA microarray, we identified 122 upregulated and 475 downregulated lncRNAs that might be related to the breast cancer chemoresistance. Among them, RP11-70C1.3 was one of the most highly expressed lncRNAs. In breast cancer patients, high RP11-70C1.3 expression predicted poor prognosis. Knockdown of RP11-70C1.3 inhibited the multidrug resistance of breast cancer cells *in vitro* and *in vivo*. Further investigations revealed that RP11-70C1.3 functioned as a competing endogenous RNA for miR-6736-3p to increase NRP-1 expression. Notably, the rescue experiments showed that both miR-6736-3p inhibitor and NRP-1 overexpression could partly reverse the suppressive influence of RP11-70C1.3 knockdown on breast cancer chemoresistance. In conclusion, our study indicated that lncRNA RP11-70C1.3 regulated NRP-1 expression by sponging miR-6736-3p to confer chemoresistance of breast cancer cells. RP11-70C1.3 might be a potential therapeutic target to enhance the clinical efficacy of chemotherapy in breast cancer.

## INTRODUCTION

Breast cancer is the most commonly diagnosed cancer and the leading cause of cancer death among women worldwide. In 2020, about 2.3 million breast cancer cases were newly established, which surpassed lung cancer as the most commonly diagnosed cancer [1]. Breast cancer is heterogeneous and can be classified into different subtypes depending on the expression status of the hormone receptors [2]. The main treatment strategies include surgery, radiation, and chemotherapy [3]. However, patients with long-term treatment of chemotherapy frequently develop chemoresistance. Nevertheless, the molecular mechanisms responsible for the poor response to chemotherapy remain poorly understood, which have become one of the major obstacles to successful treatment.

NRP-1 encodes a transmembrane glycoprotein neuropilin-1, which contains a large N-terminal extracellular domain that can bind many ligands and various types of coreceptors [4]. Previously, we reported that NRP-1 was frequently upregulated in breast cancer and functioned as an oncogene to accelerate tumorigenesis and progression by promoting proliferation, metastasis, and stemness [5-8]. Recent studies revealed that NRP-1 could promote breast cancer cells resistance to ADM and PTX resistance through activation of ITGB3/FAK/NF-kB p65 axis and downregulation of BCRP/ABCG2 [9-11]. However, a more detailed role of NRP-1 upregulation in breast cancer pathogenesis has not been fully elucidated.

Recent advances in whole genome and transcriptome sequencing technologies have identified long non-coding RNAs (lncRNAs) as a newly subgroup of non-coding RNAs [12]. LncRNAs have been shown to regulate oncogenes or tumor suppressor genes at both post-transcriptional and transcriptional level [13]. Intriguingly, lncRNAs play important roles in various biological processes, including drug resistance [14]. For example, lncRNA DILA1 promoted tamoxifen resistance of breast cancer cells through stabilizing Cyclin D1 protein [15]. Moreover, lncRNA HCP5 facilitated triple-negative breast cancer cell resistance to cisplatin by regulating PTEN expression [16]. LncRNA SNHG14 contributed to trastuzumab resistance and PABPC1 expression through regulating H3K27 acetylation in the promoter of PABPC1 [17]. Thus, understanding the expression profile and function of individual lncRNA will help to clarify the mechanisms underlying breast cancer chemoresistance.

To identify the candidate lncRNAs responsible for breast cancer chemoresistance, we first conducted an lncRNA microarray profiling analysis in three chemotherapy-resistant and three chemotherapy-sensitive breast cancer tissues. A total of 597 lncRNAs were differentially expressed, including 122 upregulated and 475 downregulated. We further identified one of the most upregulated lncRNAs RP11-70C1.3, located in chromosome 3:42876736-42893917, as a prognostic biomarker associated with a worse overall survival. The effects of RP11-70C1.3 on breast cancer cell chemoresistance were evaluated by loss-of-function analysis *in vitro* and *in vivo*. Moreover, we explored the mechanism underlying the biological role of RP11-70C1.3 in which RP11-70C1.3 functioned as a competing endogenous RNA (ceRNA) of miR-6736-3p to upregulate NRP-1 expression. Taken together, these results provide new insights into the key role of the lncRNA RP11-70C1.3 in breast cancer chemoresistance.

## MATERIALS AND METHODS

### Clinical specimens

Thirty-two chemotherapy-resistant breast cancer tissues and 28 chemotherapy-sensitive breast cancer tissues were collected from diagnosed patients who underwent surgical resection or biopsy between April 2008 and March 2013 at the Second Affiliated Hospital of Xuzhou Medical University. All enrolled patients received preoperative chemotherapy (36% adjuvant anthracycline-based therapy, 44% anthracyclines in combination with taxanes, and 20% other chemotherapy agents). All patients were signed with informed consent and the project protocol was approved by the Ethics Committee of the Second Affiliated Hospital of Xuzhou Medical University.

### Cell culture and transfection

Human breast cancer cell lines MDA-MB-231, ZR-75-1, MDA-MB-468, BT-474, MCF-7, and a normal breast epithelial cell line MCF-10A were obtained from the American Type Culture Collection. All cell lines were cultured in RPMI 1640 medium (Gibco) supplemented with 10% fetal bovine serum (FBS; Gibco) in a humidified 5% CO_2_ atmosphere at 37°C. The small interfering RNAs (siRNAs; si-NC, si-RP11-70C1.3), short hairpin RNA (shRNA; sh-NC, sh-RP11-70C1.3), miR-6736-3p mimics, miR-6736-3p inhibitor, miR-NC, and overexpression plasmids (pcDNA3.1-NC, pcDNA3.1-NRP-1) were synthesized by RiboBio (Guangdong, China). Transfections were conducted with 50 nM oligonucleotides or 100 nM plasmids using lipofectamine RNAiMAX (Invitrogen). Cells were harvested 24 or 48 h after transfection for further study.

### RNA extraction and quantitative real-time polymerase chain reaction (qRT-PCR)

Total RNA was extracted using the RNeasy mini kit (Qiagen) and cDNA was synthesized with the TIANScript II RT Kit (Qiagen). Gene expression was assessed by qRT-PCR using SYBR Premix Dimer Eraser (Perfect Real Time) assay kits on LightCycler 480 (Roche, Basel, Switzerland). Relative quantitation was calculated using the 2^-DDCt^ method and normalized to b-actin or U6. The primers used in this study were synthesized by Sangon Biotech (Shanghai, China): RP11-70C1.3: 5’-CTGCTGCAGAAGGGAGATTC-3’(forward); 5’-ACGCATGTGGATCAGATGAA-3’(reverse);miR-6736-3p: 5’-GCGGCGGTCAGCTCCTCTCTAC-3’(forward); 5’-ATCCAGTGCAGGGTCCGAGG-3’(reverse);NRP-1: 5’-GCGAAGTCTTTTGAGGGCAA-3’(forward); 5’-GCCTGGTCGTCATCACATTC-3’ (reverse).

### Establishment of chemoresistant breast cancer cell lines

Adriamycin (ADM)-resistant cells MB231/ADM and paclitaxel (PTX)-resistant cells MCF-7/PTX were constructed by treatment of MCF-7 cells or MDA-MB-231 with stepwise increasing concentrations of ADM or PTX until they acquired resistance to 5 mM ADM or 20 mM PTX (Sigma), respectively. The resistant cells were routinely maintained in the presence of 5 mM ADM or 20 mM PTX every other day and are removed before the experiments being performed.

### Colony formation assay

Cells were seeded in 6-well plates at a density of 500 cells per well. After 2 weeks, the cells were washed 2 times with PBS, fixed with methanol for 30 min, and stained with 0.1% crystal violet (Beyotime, Shanghai, China) for 15 min. The number of visible colonies was counted under a microscope.

### Drug resistance assay

The breast cancer cells (5 × 10^3^ per well) were seeded in 96-well plates. After cellular adhesion, cells were incubated with freshly prepared anticancer drugs (Sigma) including ADM, PTX, cisplatin (DDP), cyclophosphamide (CPM), fluorouracil (5-Fu), vincristine (VCR), and methotrexate (MTX) for 48 h. Subsequently, 10 mL CCK-8 solution (Beyotime) was added to each well. After 1 h of incubation at 37°C, the optical density (OD) at 450 nm was measured under a microplate reader (Thermo Fisher Scientific). The half-maximal inhibitory concentration (IC_50_) value of anticancer drugs was analyzed by the relative survival curve to assess the cell resistance. The resistance index was calculated as follows: Resistance index = IC50 (resistant cells)/IC50 (control cells). The original IC50 values of each cell line of different anticancer drugs and related resistance index are shown in Supplementary Tables 1-5.

### RNA pull-down assay

Biotinylated at 3’ ends of miR-6736-3p (Bio-miR-6736-3p) and miR-NC (Bio-miR-NC) were synthesized by RiboBio and transfected into MB231/ADM and MCF-7/PTX cells at a concentration of 50 nM. After 48 h incubation, cells were harvested and lysed with a lysis buffer. The freshly prepared cellular lysates containing Biotinylated miRNAs were incubated with streptavidin magnetic beads (Invitrogen) for 1 h at room temperature. After washing, the NRP-1 expression was measured by qRT-PCR assay.

### Microarray analysis

The total RNA from three chemotherapy-resistant breast cancer tissues and three chemotherapy-sensitive breast cancer tissues was extracted by Trizol reagent (Invitrogen) and used to double-stranded cDNA. cDNA was synthesized, labeled, and hybridized to microarray (Arraystar 8*60K Human LncRNA Array V5.0, Aksomics Inc., Shanghai, China). The data were extracted and analyzed using GeneSpring software V11 (Agilent Technologies, Santa Clara, CA, USA). The threshold for differentially expressed lncRNAs was set as a fold change >= 2.0 and p <0.05.

### Apoptosis assay

The Annexin V-FITC/Propidium-Iodide (PI) Apoptosis Detection kit (Invitrogen) was utilized to determine cell apoptosis. Briefly, cells were harvested after transfection for 48 h and washed with phosphate-buffered solution twice. Then, these cells were stained with Annexin V-FITC and PI for 15 min at room temperature, and then immediately analyzed with BD FACSCalibur flow cytometer (BD Biosciences, Franklin Lakes, NJ).

### Western blot analysis

Protein extracts were prepared at 4°C in RIPA buffer (Beyotime), separated by 10% SDS-polyacrylamide gel electrophoresis, and transferred onto polyvinylidene difluoride membranes (BioRad, Berkeley, CA, USA). Then, the membranes were probed with primary antibodies against NRP-1 and b-actin (Abcam), at 4°C overnight followed by incubation with peroxidase-conjugated secondary antibody (Proteintech, Chicago, IL, USA). The signals were visualized by a ECL kit (GE) on MYECL Imager (Thermo).

### Mice experiments

All animal experiments were performed in accordance with the protocols approved by the Institutional Animal Care and Use Committee of the Second Affiliated Hospital of Xuzhou Medical University. MB231/ADM or MCF-7/PTX cells (2 × 10^6^) stably expressing with sh-RP11-70C1.3 or sh-NC were subcutaneously injected into right flank area of BALB/c nude mice (Vital River, Beijing, China). Animals were randomly grouped (n = 6 per group). After 7 days, mice were treated with or without 5 mg/kg ADM or 20 mg/kg PTX through intraperitoneal injections twice a week until the end of the study. Tumor volume was calculated by the following formula: Volume = (length × width^2^) × 0.5. After 5 weeks, the mice were killed and the tumor weight was recorded.

### Immunohistochemical staining

Tumor tissues were fixed in 10% formaldehyde in PBS, embedded in paraffin, and cut into 4 mm thick slices. Then, the slices were deparaffinized with xylene, rehydrated, and microwaved for antigen retrieval with a citrate buffer using routine methods. The tissue sections were blocked with 1% bovine serum albumin buffer for 20 min at room temperature, incubated with the primary antibodies against Ki-67 (Cell Signaling Technology, 1:50) overnight at 4°C, and then treated with biotin-labeled secondary antibodies for 30 min at room temperature, followed by staining with diaminobenzidine, counterstaining with 10% Mayer’s hematoxylin, dehydration, and mounting.

### Luciferase reporter assay

The wide type (wt) or mutated (mut) response elements of miR-6736-3p in the RP11-70C1.3 and 3’UTR of NRP-1 were synthesized and cloned into pMIR-REPORT plasmid downstream of luciferase reporter gene. Then, wt- or mut- reporters and miR-6736-3p mimics or negative control were cotransfected into breast cancer cells. After 48 transfection, luciferase activities were assessed by a luciferase reporter assay system (Promega) according to the manufacturer’s instruction.

### Statistical analysis

The mean values ± S.D. were calculated and plotted using GraphPad Prism 9 software (GraphPad Software). Comparisons were analyzed by the one-way analysis of variance (ANOVA) followed by Bonferroni *post hoc* test or Student’s t-test. In addition, Kaplan–Meier survival curves were plotted, and a log-rank test was performed. Differences were defined as statistically significant for p < 0.05.

## RESULTS

### LncRNA RP11-70C1.3 is ectopically overexpressed in the chemoresistant breast cancer tissues and associated with poor overall survival

**FIGURE 1:**
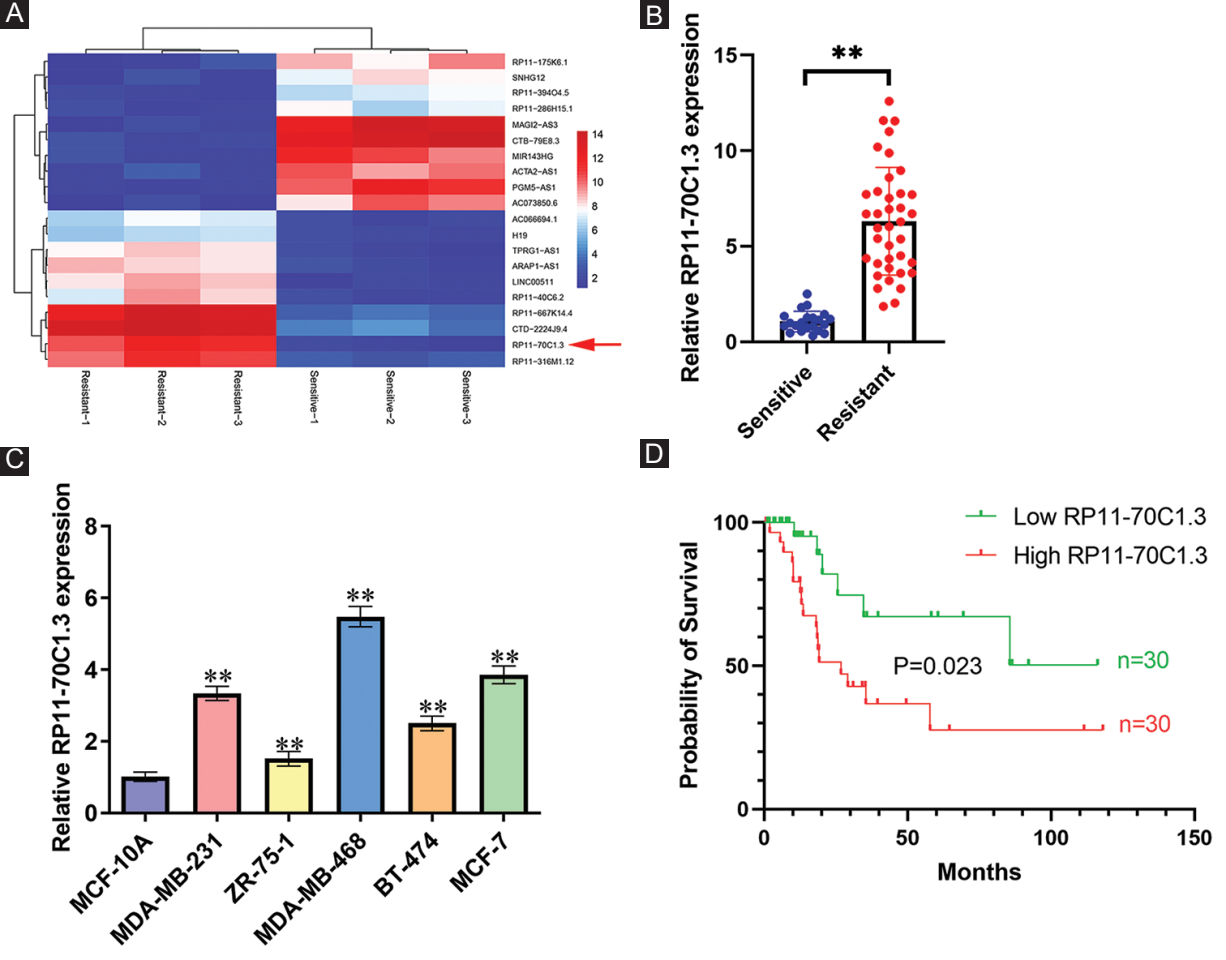
LncRNA RP11-70C1.3 is ectopically overexpressed in the chemoresistant breast cancer tissues and associated with poor overall survival. (A) Heat map of top 10 upregulated and 10 downregulated lncRNAs in three chemotherapy-resistant and three chemotherapy-sensitive breast cancer tissues. (B) Relative RP11-70C1.3 expression in 32 chemotherapy-resistant tissues and 28 chemotherapy-sensitive breast cancer tissues, as detected by qRT-PCR. (C) Relative RP11-70C1.3 expression in five human breast cancer cell lines and a normal breast epithelial cell line MCF-10A, as detected by qRT-PCR. (D) Kaplan–Meier survival curves for breast cancer patients with high and low expression of RP11-70C1.3. **p < 0.01.

To explore the roles of lncRNAs in breast cancer chemoresistance, an lncRNA microarray analysis was performed in three chemotherapy-resistant and three chemotherapy-sensitive breast cancer tissues. A total of 122 upregulated and 475 downregulated lncRNAs were identified (Figure 1A). Among them, lncRNA RP11-70C1.3 was one of the most highly expressed lncRNAs in chemoresistant breast cancer tissues. qRT-PCR results validated that RP11-70C1.3 expression was strikingly increased 6.32-fold compared with chemosensitive breast tissues (Figure 1B). The expression of RP11-70C1.3 was further confirmed in five breast cancer cell lines and a normal breast epithelial cell line MCF-10A using qRT-PCR. Undoubtedly, compared with MCF-10A cells, RP11-70C1.3 expression was significantly increased in breast cancer cell lines (Figure 1C). Kaplan–Meier survival analysis and log-rank tests demonstrated that patients with higher expression level of RP11-70C1.3 had a poor overall survival (*p* = 0.023; Figure 1D). Furthermore, we observed that there was a strong correlation of RP11-70C1.3 expression with distant metastasis and clinical stage in breast cancer patients (Table 1). These results indicate that lncRNA RP11-70C1.3 might be an important regulator of breast cancer chemoresistance.

**TABLE 1:**
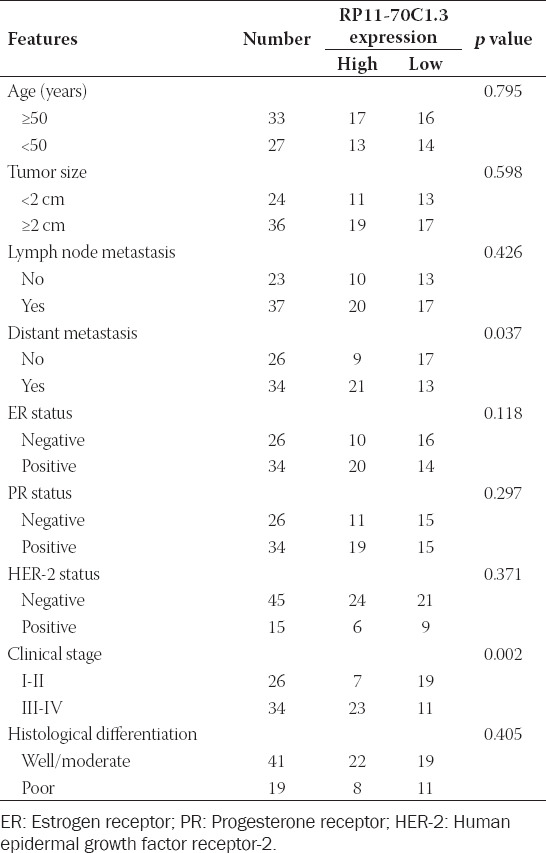
Correlation of RP11-70C1.3 expression and clinicopathological features of breast cancer patients

### RP11-70C1.3 knockdown suppresses the chemoresistance of breast cancer cells *in vitro*

**FIGURE 2:**
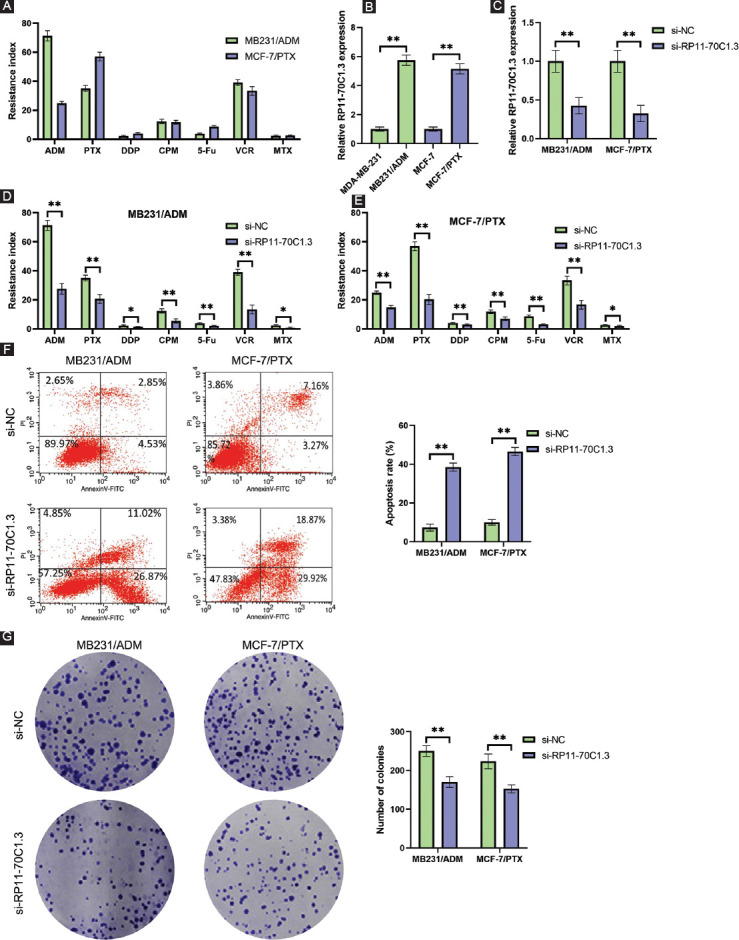
RP11-70C1.3 knockdown suppresses the chemoresistance of breast cancer cells in vitro. (A) Resistance index of MB231/ADM and MCF-7/PTX cells for ADM, PTX, DDP, CPM, 5-Fu, VCR, and MTX, as detected by CCK-8 assay. (B) Relative RP11-70C1.3 expression in MB231/ADM and MCF-7/PTX cells and their parental cells, as detected by qRT-PCR. (C) Relative RP11-70C1.3 expression in MB231/ADM and MCF-7/PTX cells after RP11-70C1.3 knockdown, as detected by qRT-PCR. (D) and (E) Resistance index of MB231/ADM (D) and MCF-7/PTX (E) cells for ADM, PTX, DDP, CPM, 5-Fu, VCR, and MTX in MB231/ADM and MCF-7/PTX cells after RP11-70C1.3 knockdown, as detected by CCK-8 assay. (F) RP11-70C1.3 knockdown promoted apoptosis of MB231/ADM and MCF-7/PTX cells compared with si-NC, as detected by flow cytometry assay. (G) RP11-70C1.3 knockdown reduced the capability of colony formation in MB231/ADM and MCF-7/PTX cells, as detected by colony formation assay. *p < 0.05; **p < 0.01.

To confirm the association of RP11-70C1.3 expression and breast cancer chemoresistance, ADM-resistant cells MB231/ADM and PTX-resistant cells MCF-7/PTX were constructed (Figure 2A). qRT-PCR results demonstrated that RP11-70C1.3 expression was markedly increased in resistant cells compared with parental cells (Figure 2B). Next, we determined the effects of RP11-70C1.3 knockdown on the cell chemoresistance by transfecting si-RP11-70C1.3 or si-NC into MB231/ADM and MCF-7/PTX cells (Figure 2C). CCK-8 assays results showed that RP11-70C1.3 knockdown significantly sensitized resistant cells to chemotherapy drugs (Figure 2D and E). In both MB231/ADM and MCF-7/PTX cells, we observed lower colony numbers but a higher apoptosis rate after RP11-70C1.3 silence (Figure 2F and G). Together, the evidence in this section suggests that RP11-70C1.3 knockdown inhibited breast cancer chemoresistance.

### RP11-70C1.3 knockdown inhibits breast cancer chemoresistance *in vivo*

**FIGURE 3:**
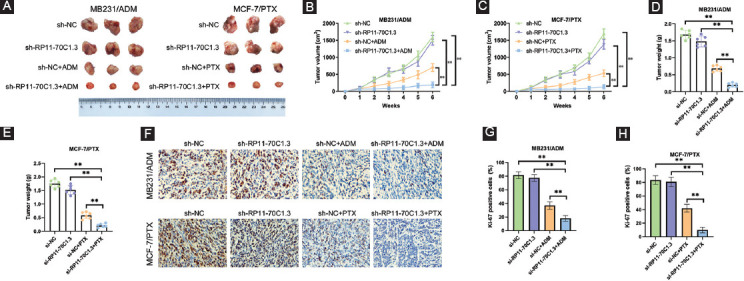
RP11-70C1.3 knockdown inhibits breast cancer chemoresistance *in vivo*. Stably expressing sh-RP11-70C1.3 or sh-NC MB231/ADM and MCF-7/PTX cells were subcutaneously injected into the right flank area of nude mice. After 7 days, mice were treated with or without 5 mg/kg ADM or 20 mg/kg PTX through intraperitoneal injections twice a week until the end of the study. Five weeks later, the mice were killed and the tumor weight was recorded. (A) Representative pictures of tumor tissues. (B) and (C) Tumor volume from MB231/ADM (B) and MCF-7/PTX (C) cells. (D) and (E) Tumor weight from MB231/ADM (D) and MCF-7/PTX (E) cells. (F) Ki-67 immunohistochemical staining analysis. (G) and (H) Statistical analysis of Ki-67 positive cells from MB231/ADM (G) and MCF-7/PTX (H) cells. **p < 0.01.

To further evaluate the influence of RP11-70C1.3 on breast cancer chemoresistance, *in vivo* xenograft assay was performed by injecting stably expressing sh-RP11-70C1.3 or sh-NC-resistant cells subcutaneously into right flank area of nude mice and then treated with or without chemotherapeutic agents (Figure 3A). Our results demonstrated that RP11-70C1.3 knockdown could significantly reduce the tumor growth (Figure 3B and C) and weight (Figure 3D and E) in mice treated with chemotherapeutic agents. However, there was no statistical difference of tumor growth and weight between sh-RP11-70C1.3 group and sh-NC group without chemotherapeutic agents’ treatment. Immunohistochemical staining demonstrated that Ki-67-positive cells in the sh-RP11-70C1.3 group were lower than that in the sh-NC group (Figure 3F-H). These findings consolidated the crucial role of RP11-70C1.3 in breast cancer chemoresistance.

### RP11-70C1.3 is a sponge of miR-6736-3p

**FIGURE 4:**
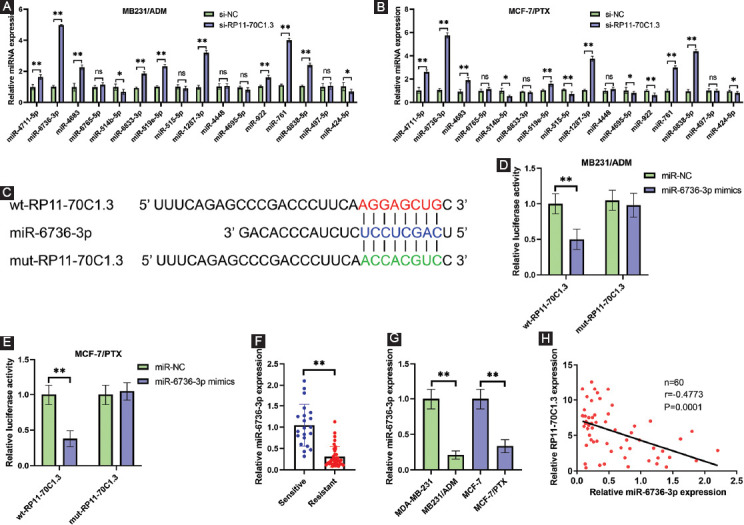
RP11-70C1.3 is a sponge of miR-6736-3p. (A) and (B) DIANA-LncBase analysis revealed that 16 candidate miRNAs could interact with RP11-70C1.3. qRT-PCR was used to determine their expression levels in MB231/ADM (A) and MCF-7/PTX (B) cells after RP11-70C1.3 knockdown. (C) Putative binding site of miR-6736-3p for RP11-70C1.3. (D) and (E) Luciferase reporter assay results showed that miR-6736-3p mimics reduced the luciferase activity of wt-RP11-70C1.3 but not that of the mut-RP11-70C1.3 vector in MB231/ADM (D) and MCF-7/PTX (E) cells. (F) Relative miR-6736-3p expression in 32 chemotherapy-resistant tissues and 28 chemotherapy-sensitive breast cancer tissues, as detected by qRT-PCR. (G) Relative miR-6736-3p expression in MB231/ADM and MCF-7/PTX cells and their parental cells, as detected by qRT-PCR. (H) Pearson correlation analysis demonstrated that miR-6736-3p negatively correlated with RP11-70C1.3 expression in breast cancer tissues. ns *p* > 0.05; * p < 0.05; ** *p* < 0.01.

To uncover the possible mechanism through which RP11-70C1.3 regulated breast cancer chemoresistance, the online bioinformatics tool DIANA-LncBase was used to predict the candidate miRNAs that might interact with RP11-70C1.3 [18]. Among the 16 miRNAs, the qRT-PCR results revealed that miR-6736-3p was the most upregulated miRNA after RP11-70C1.3 knockdown in both MB231/ADM and MCF-7/PTX cells (Figure 4A and B). To substantiate a direct interaction between RP11-70C1.3 and miR-6736-3p, wt- and mut-RP11-70C1.3 reporter containing the putative binding sites for miR-6736-3p was constructed (Figure 4C). Luciferase reporter assays results demonstrated that miR-6736-3p mimics suppressed the luciferase activity of the wt-RP11-70C1.3 but not that of the mut-RP11-70C1.3 vector (Figure 4D and E). Furthermore, we detected the expression level of miR-6736-3p in chemoresistant breast cancer tissues and cell lines. We observed a significant decrease of miR-6736-3p expression in chemoresistant breast cancer tissues in comparison with chemosensitive cases (Figure 4F). Similarly, miR-6736-3p expression was also strikingly reduced in MB231/ADM and MCF-7/PTX cells (Figure 4G). As shown in Figure 4H, there was a significant negative correlation between miR-6736-3p and RP11-70C1.3 expression in breast cancer tissues (r= −0.4773, P=0.0001). These results suggest that miR-6736-3p may be a downstream target of RP11-70C1.3.

### miR-6736-3p directly targets NRP-1 and represses its expression

**FIGURE 5:**
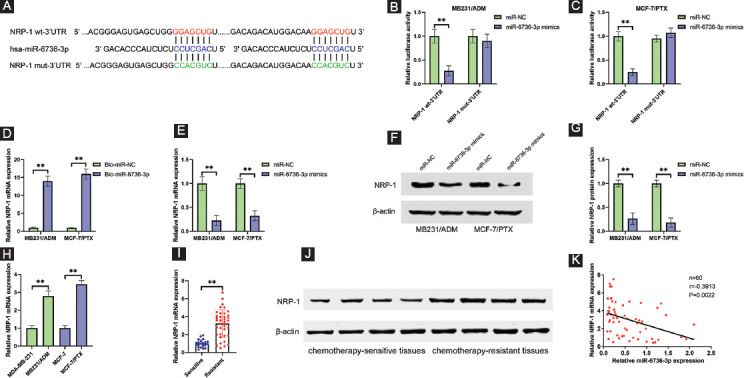
miR-6736-3p directly targets NRP-1 and represses its expression. (A) Predicted binding sites of miR-6736-3p for the 3’UTR of NRP-1, as analyzed by TargetScan v7.2. (B) and (C) Luciferase reporter assay results showed that miR-6736-3p mimics reduced the luciferase activity of NRP-1 wt-3’UTR but not that of the NRP-1 mut-3’UTR vector in MB231/ADM (B) and MCF-7/PTX (C) cells. (D) Biotinylated miR-6736-3p and miR-NC were transfected into MB231/ADM and MCF-7/PTX cells. RNA pull-down assay results showed that NRP-1 mRNA was highly enriched in Bio-miR-6736-3p group. (E) Effects of miR-6736-3p overexpression on the mRNA levels of NRP-1 in MB231/ADM and MCF-7/PTX cells determined by qRT-PCR. (F) Effects of miR-6736-3p overexpression on the protein levels of NRP-1 in MB231/ADM and MCF-7/PTX cells were determined by Western blot. (G) Quantitative analysis of Figure 5F. (H) Relative NRP-1 mRNA expression in MB231/ADM and MCF-7/PTX cells and their parental cells, as detected by qRT-PCR. (I) and (J) Relative NRP-1 mRNA and protein expression in 32 chemotherapy-resistant tissues and 28 chemotherapy-sensitive breast cancer tissues, as detected by qRT-PCR and Western blot. (K) Pearson correlation analysis demonstrated that miR-6736-3p negatively correlated with NRP-1 mRNA expression in breast cancer tissues. ** *p* < 0.01.

Our previous studies indicated that knockdown of NRP-1 blocks cell proliferation, metastasis, stemness, and promotes cell apoptosis in breast cancer [5-8]. Therefore, we wondered whether NRP-1 is a target of miR-6736-3p. Interestingly, NRP-1 3’UTR was predicted to contain two binding sites of miR-6736-3p at position 61-67 and 165-171 using TargetScan (Figure 5A) [19]. Luciferase reporter assay results revealed that cotransfection NRP-1 wt-3’UTR vector with miR-6736-3p mimics significantly decreased luciferase activity, while there was no significant change in luciferase activity for NRP-1 mut-3’UTR vector (Figure 5B and C). RNA pull-down assay further confirmed the direct interaction between miR-6736-3p and NRP-1 (Figure 5D). In addition, miR-6736-3p overexpression obviously reduced NRP-1 mRNA and protein level in both MB231/ADM and MCF-7/PTX cells (Figure 5E-G). Meanwhile, we determined the expression of NRP-1 in chemoresistant breast cancer cells and tissues. As expected, the NRP-1 mRNA and protein levels were distinctly increased in chemoresistant cells and tissues compared with chemosensitive cells and tissues, respectively (Figure 5H-J). Moreover, NRP-1 and miR-6736-3p were negatively correlated in breast cancer tissues (Figure 5K). According to the above findings, NRP-1 might be a target of miR-6736-3p.

### RP11-70C1.3 promotes breast cancer chemoresistance through regulating miR-6736-3p/NRP-1 axis

**FIGURE 6:**
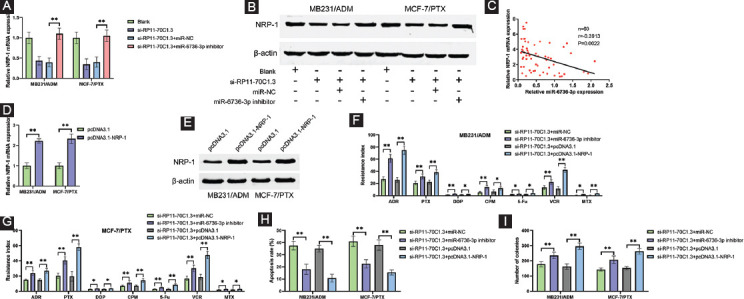
RP11-70C1.3 promotes breast cancer chemoresistance through regulating miR-6736-3p/NRP-1 axis. (A) and (B) miR-6736-3p inhibitors reversed the suppression of RP11-70C1.3 knockdown on NRP-1 mRNA (A) and protein (B) level in both MB231/ADM and MCF-7/PTX cells. (C) Pearson correlation analysis demonstrated that RP11-70C1.3 positively correlated with NRP-1 mRNA expression in breast cancer tissues. (D) and (E) qRT-PCR and Western blot were used to validate the upregulation of NRP-1 in mRNA (D) and protein (F) levels in MB231/ADM and MCF-7/PTX cells after transfected with pcDNA3.1-NRP-1 or its negative control pcDNA3.1. (F) and (G) CCK-8 assay results showed that transfection of miR-6736-3p inhibitor or pcDNA3.1-NRP-1 attenuated the chemoresistance inhibition induced by RP11-70C1.3 knockdown in MB231/ADM (F) and MCF-7/PTX (G) cells. (H) The apoptosis rate induced by RP11-70C1.3 knockdown in MB231/ADM and MCF-7/PTX cells was obviously decreased after transfection with miR-6736-3p inhibitor or pcDNA3.1-NRP-1. (I) The effects of RP11-70C1.3 knockdown on the capability of colony formation in MB231/ADM and MCF-7/PTX cells were reversed after miR-6736-3p silence or NRP-1 overexpression. * *p* < 0.05; ** *p* < 0.01.

To figure out whether RP11-70C1.3 could affect NRP-1 expression by sponging miR-6736-3p, si-RP11-70C1.3 and miR-6736-3p inhibitors were cotransfected into MB231/ADM and MCF-7/PTX cells. As shown in Figure 6A and B, RP11-70C1.3 knockdown significantly reduced NRP-1 mRNA and protein expression, while NRP-1 level was reversed after miR-6736-3p silencing. Pearson correlation analysis also showed that RP11-70C1.3 had a significantly positive correlation with NRP-1 in breast cancer tissues (Figure 6C). These data implied that RP11-70C1.3 functioned as a ceRNA to upregulate NRP-1 expression by sponging miR-6736-3p. To investigate whether RP11-70C1.3 facilitated breast cancer chemoresistance through a miR-6736-3p/NRP-1-dependent manner, we constructed NRP-1-overexpressing vector pcDNA3.1-NRP-1. qRT-PCR and Western blot were used to validate the upregulation of NRP-1 in mRNA and protein levels (Figure 6D and E). Our results showed that RP11-70C1.3 knockdown inhibited cell chemoresistance, while cotransfection with miR-6736-3p inhibitor or pcDNA3.1-NRP-1 could dramatically eliminate chemoresistance (Figure 6F and G). Consistent with these results, we also observed that the effects of RP11-70C1.3 knockdown on cell colony formation and apoptosis were substantially reversed by miR-6736-3p downregulation or NRP-1 overexpression (Figure 6H and I). Collectively, our data strongly support the claim that RP11-70C1.3 positively regulates breast cancer chemoresistance through miR-6736-3p/NRP-1 axis.

## DISCUSSION

At present, *de novo* or acquired resistance of breast cancer cells to chemotherapeutic drugs is particularly relevant to recurrence and metastasis, leading to a poor prognosis [20-22]. In this study, we focused on the molecular mechanism of breast cancer chemoresistance, with a particular interest in lncRNAs, which have been broadly implicated in several types of human tumor [23].

Based on our lncRNA microarray result, we identified 597 differentially expressed lncRNAs that might be related to the development of drug resistance in breast cancer. Among them, RP11-70C1.3 was one of the most highly expressed lncRNAs and associated with an unfavorable prognosis. Interestingly, we found that high expression level of RP11-70C1.3 significantly correlated with distant metastasis, indicating that RP11-70C1.3 might also play an important role in regulating breast cancer cell metastasis. Through the loss-of-function assays *in vitro* and *in vivo*, we discovered that knockdown of RP11-70C1.3 sensitized breast cancer cells to a series of chemotherapeutic drugs, including PTX, DDP, ADM, CPM, 5-Fu, VCR, and MTX, however, the underlying mechanism was unclear.

In accordance with the ceRNA hypothesis, lncRNA transcripts can function as endogenous decoys for miRNAs through their miRNA binding sites [24-26]. Bioinformatics analysis revealed that several miRNAs were candidate downstream targets of RP11-70C1.3. Among them, miR-6736-3p was the most downregulated miRNA after RP11-70C1.3 knockdown. Luciferase reporter assays further validated the direct interaction between RP11-70C1.3 and miR-6736-3p. Moreover, miR-6736-3p was lowly expressed in chemoresistant breast cancer tissues and cell lines, which was different from a recent report of miR-6736-3p upregulating in gastric cancer [27]. However, the biological role of miR-6736-3p in tumorigenesis and development has not been investigated. In addition, miR-6736-3p showed a negative correlation with RP11-70C1.3 in our enrolled 60 breast cancer tissues. Taken together, these findings indicated that miR-6736-3p was a downstream target of RP11-70C1.3.

Growing evidence has demonstrated that NRP-1 was frequently overexpressed and associated with poor prognosis in gastric cancer, melanoma, bladder cancer, cervical cancer, hepatocellular carcinoma, as well as breast cancer [28-33]. Our previous studies demonstrated that NRP-1 acts as an oncogene in breast cancer progression [5-8]. Furthermore, recent evidence showed a close association of NRP-1 and breast cancer chemoresistance [9-11]. Collectively, the above mentioned findings indicate that NRP-1 is a crucial regulator in breast cancer pathogenesis and might be responsible for the chemoresistance medicated by RP11-70C1.3/miR-6736-3p.

In this study, NRP-1 3’UTR was predicted to contain two binding sites of miR-6736-3p. Further investigations demonstrated that NRP-1 was a direct target of miR-6736-3p, and RP11-70C1.3 functioned as a sponge of miR-6736-3p to upregulate NRP-1 expression. Consistent with the previous studies, we observed an increased expression of NRP-1 in breast cancer chemoresistant tissues and cell lines. Besides, NRP-1 positively correlated RP11-70C1.3 but negatively correlated miR-6736-3p. More importantly, rescue experiments showed that NRP-1 overexpression reversed the inhibitory effects of RP11-70C1.3 knockdown on breast cancer cell chemoresistance. Taken together, our data support an unequivocal role for RP11-70C1.3/miR-6736-3p/NRP-1 axis in the development of breast cancer chemoresistance.

## CONCLUSION

Our data showed that RP11-70C1.3 promoted chemoresistance of breast cancer cells *in vivo* and *in vitro*. Furthermore, we established a novel mechanism that RP11-70C1.3 functioned as a molecular sponge for miR-6736-3p and upregulated its target NRP-1 expression. Overall, RP11-70C1.3 may be a potential therapeutic target for breast cancer treatment.

## Figures and Tables

**SUPPLEMENTARY TABLE 1:**
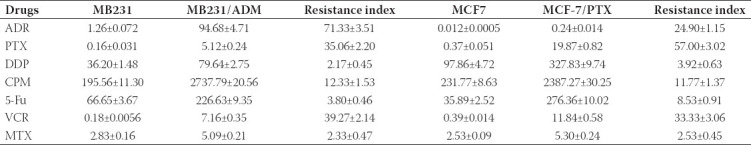
The IC50 values of each drug for MB231, MB231/ADM, MCF7, and MCF-7/PTX cells

**SUPPLEMENTARY TABLE 2:**
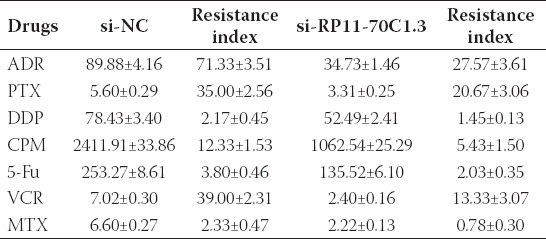
The IC50 values of each drug for MB231/ADM cells after transfected with si-NC or si-RP11-70C1.3

**SUPPLEMENTARY TABLE 3:**
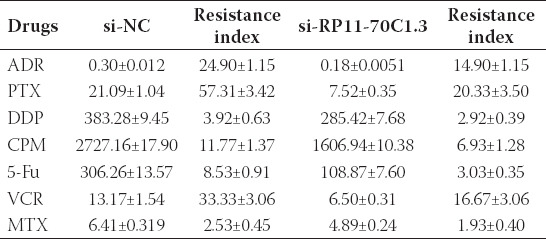
The IC50 values of each drug for MCF-7/PTX cells after transfected with si-NC or si-RP11-70C1.3

**SUPPLEMENTARY TABLE 4:**
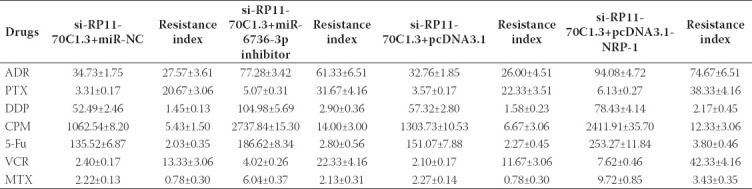
The IC50 values of each drug for MB231/ADM cells after cotransfected si-RP11-70C1.3 with miR-NC, miR-6736-3p inhibitor, pcDNA3.1, or pcDNA3.1-NRP-1

**SUPPLEMENTARY TABLE 5:**
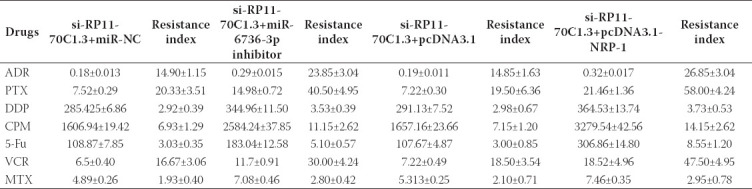
The IC50 values of each drug for MCF-7/PTX cells after cotransfected si-RP11-70C1.3 with miR-NC, miR-6736-3p inhibitor, pcDNA3.1, or pcDNA3.1-NRP-1
